# How to Compare Relative Age Effect in Different Sports? A New Methodological Approach—Example of Youth Olympic Games

**DOI:** 10.3390/sports12080215

**Published:** 2024-08-08

**Authors:** Drazen Cular, Matej Babic, Darko Katovic, Tea Beslija, Ana Kezic

**Affiliations:** 1 Faculty of Kinesiology, University of Split, N. Tesle 6, 21000 Split, Croatia; drazen.cular@kifst.eu (D.C.); matej.babic@kifst.eu (M.B.); tea.beslija@hotmail.com (T.B.); 2 Einstein, Startup for Research, Development, Education, Trade and Services, M. Krleze 12, 21000 Split, Croatia; 3 European Institute for Talents, Education, Research & Development, M. Krleze 12, 21000 Split, Croatia; 4 Faculty of Kinesiology, University of Zagreb, Horvacanski zavoj 15, 10000 Zagreb, Croatia; darko.katovic@kif.unizg.hr

**Keywords:** basketball, handball, swimming, taekwondo, talent detection, RAE, age quartiles

## Abstract

This research aimed to propose a new methodological approach for analyzing relative age effect (RAE) in different sports or samples named “Relative age effect overall scale” (RAEOS). The sample consisted of 1455 male and female young athletes who competed in four different sports (basketball, *n* = 159; handball, *n* = 215; swimming, *n* = 981; taekwondo, *n* = 100) at the Youth Olympic Games (YOG) in Buenos Aires in 2018. To construct the new model, the sample was classified into four unified quartiles of a specific range depending on the sport (swimming: 48-month range, taekwondo: 24-month range, and basketball and handball: 36-month range). Expected and observed frequencies for each sport, the winners/all athletes, and differences between team and individual sports were analyzed using a non-parametric Chi-square test. The obtained results confirm the existence of the RAE in all four analyzed sports (*p* > 0.01) in a sample of all participants and the sample of gold medalists. Differences between team and individual sports in the analyzed sample have also been found. The proposed methodological approach (RAEOS) is a simple and applicable tool that provides opportunities for comparison and analysis of different sports and competition formats, as well as improvement of the sports talent system in the context of RAE issues. It is suggested to the sports decision-makers to improve the YOG qualification and competition system to enable fairer competition and reduce the influence of RAE on the performance and development of young athletes.

## 1. Introduction

Nowadays, youth sports are receiving greater public attention, as the lifestyle of children has become more sedentary due to the fourth industrial revolution. The benefits of sustained physical activity for the health of individuals, regardless of age, have long been known and promoted [1,2]. Due to enhanced public interest, questions of talent identification, sports careers, and fair conditions in youth sports became important and interesting to coaches, parents, and scientists. Scientists found various effects that may impact the sports careers of children and youth. Hancock, Adler, and Cote [3] proposed a theoretical model in which the authors explained the impact of the Matthew, Plygamon, and Galatea effects, and their interaction with the relative age effect (RAE). The Matthew effect relates to how parents’ perceptions of their child’s physical attributes influence their choice of sports. The Pygmalion effect suggests that higher expectations lead to greater achievements, often based on physical maturity rather than skill. The Galatea Effect mirrors the Pygmalion effect, showing how individuals align their beliefs and actions with external expectations, sometimes to their detriment. What the later authors propose is that parents influence RAEs through Matthew effects, coaches influence RAEs through Pygmalion effects, and athletes influence RAEs through Galatea effects.

The relative age effect is a phenomenon that occurs in almost all sports competitions and gives an advantage to those born earlier in the same year and from the same category [4]. The effects of the relative age work over a longer period, leading to what is known as the constituent year effect (CYE) [5,6,7]. Although an age difference of less than 12 months may have little relevance for adults, it may be significant in children [8]. RAE is likely to manifest during the period of adolescent growth spurt, characterized by significant variability in growth and maturation among individuals, which yields to accelerant individuals and goes against stagnant individuals. It is presumed that at a younger age and following the growth spurt, the RAE diminishes as growth rates become more uniform and slower, particularly as late-maturing individuals reach adulthood.

Talent identification systems are based on selection biases that confuse maturation for talent [9]. Processes of initiation, identification, and selection are very important but hazardous procedures, and if not conducted correctly, they lead to talent dispersal [10]. Relative age is the term that has frequently been investigated over the past two decades, and much evidence has been collected about the influence of RAE in sports. For instance, Pino-Ortega et al. [11] suggest that RAE occurs even within different player positions and affects overall (kinematic) performance parameters. Moreover, Faber et al. [12] detected the existence of RAE within years and between years among elite competitors. Recent studies have explored various aspects of RAE and proposed new models and methods to understand and mitigate its effects. For instance, research by Pedersen et al. [13] in the context of elite youth soccer has shown that RAE varies with age and sex. It is generally stronger in male athletes and in sports with higher competition levels. This study also highlights the need for a nuanced approach to understanding RAE, considering factors such as the sport type, age group, and level of competition. Smith et al. [14] conducted a systematic review and meta-analysis on RAE across various female sports contexts. They concluded that while RAE is generally weaker in female sports compared to male sports, it still exists and can influence talent identification and development. This review provided insights into the variability of RAE across different sports and age groups. Authors [15] have examined the RAE in the Australian football talent pathway. They found that RAE significantly influences the progression from entry-level to elite-level participation, with relatively older athletes being more likely to be identified as talented and selected for higher levels of competition.

In the context of scientific work, there are some existing biases about the methods of RAE determination, as well as about the comparison of different (individual and team) sports and age categories. Every sport has its chronological age boundaries within every age category, and speaking of different sports, their age boundaries within categories also differ. For example, some sports have a range of three years within the cadet age category, while other sports have a two- or one-year span. Further, even in the same sport (e.g., taekwondo), there is a different age range within the same age categories between different competition levels, where the cadet category has a three-year span at the national level, but a two-year span at the international level (e.g., Youth Olympic Games). Considering the authors’ literature review on this topic, there is no defined methodological approach that successfully compares different sports and age categories yet. Previous investigations about RAE, especially systematic reviews [16,17] and meta-analyses [18], have shown a methodological deficiency in the comparison of different sample grouping methods, respectively, between semesters (S1, S2) and quartiles (Q1, Q2, Q3, Q4) within one-, two-, or three-year spans. Besides studying RAE, which is based on the date of birth, scientists are increasingly engaged in developing various methods and modern technologies [19] and combinations of methodological approaches [20] that aim to improve existing competition systems based on biological age and dependent on age categories. Also, categorization systems are typically conducted solely based on chronological age.

Accordingly, there is a growing need for a more advanced methodological approach that could answer the RAE comparison within the wide spectrum of sports, disciplines, and age categories. Such a system would enhance the knowledge about RAE, help scientists conduct more extensive analyses, and improve sports talent systems and talent support. Therefore, this research aimed to investigate the presence of RAE in different sports by proposing a new methodological approach to standardize such differently presented records between different sports named “Relative age effect overall scale” (RAEOS).

## 2. Materials and Methods

### 2.1. Participants

The sample consisted of 1455 young male and female athletes who competed in four sports (basketball, *n* = 159; handball, *n* = 215; swimming, *n* = 981; taekwondo, *n* = 100) at the Youth Olympic Games in Buenos Aires 2018 (YOG). Birth dates of athletes were collected from the official web page of the Olympic World Library (https://library.olympics.com/Default/doc/SYRACUSE/177522/official-results-books-buenos-aires-2018-youth-olympic-games-buenos-aires-youth-olympic-games-organi?_lg=en-GB, accessed on 19 March 2020). The criterion for including athletes in the sample was to select all athletes competing at the YOG and to include two individual and two team sports that encompass varying age spans (ranging from two to four years).

### 2.2. Study Design

Each of the four sports has specific rules, qualification methods, and age ranges for participation in the YOG. Given that the age ranges of the athletes who competed at the YOG include two (taekwondo), three (basketball and handball), and four (swimming) years, to construct a new model, the sample has been divided into four quartiles specific to each of the sports (Table 1) in a way that each quartile covered a different age range for each sport. The method of identifying the ranks (the number of months divided by the 4 quarters) was used in this descriptive research. Therefore, each age span (per sport) in months was divided by four in order to obtain the range of months for each of the quartiles. For example, in swimming, where athletes compete within a 4-year age span (48 months), the first quartile represented those who were born from the 1st month to the 12th month of the year 2000 (first 12 months), while in taekwondo, where athletes compete within a 2-year age span (24 months), the first quartile represented those born from the 1st month to the 6th month of the year 2001 (6 months). In this way, all athletes were categorized into 4 categories in a range that is unique to each sport. This approach is the only way to enable comparisons across different sports and investigate the presence of the RAE among them.

### 2.3. Statistical Analysis

Statistical data processing was carried out using the analytical tool Microsoft Excel for spreadsheet calculations, which is an integral part of the Microsoft Office software package (version Microsoft Office Professional Plus 2019), and the open-source graphical user interface for the R programming language R jamovi (Version 4.1). The Non-parametric Chi-Square Test (Goodness of Fit and Test of Independence) was used to detect possible differences between expected and observed frequencies for each sport, and differences between team and individual sports. A statistical level of significance of 99% (*p* < 0.01) was applied.

## 3. Results

The construction of a new methodological approach, RAEOS, based on age quartiles, separately for each sport, enabled the calculation of the relative age effect within each sport, and the results are presented in Table 2. The expected frequences per quartile (EFPQ) were derived from the sum of all observed frequences (total number of participants per sport) by dividing it by four (it was expected that each age quartile would have the same number of participants).

**Figure 1:**
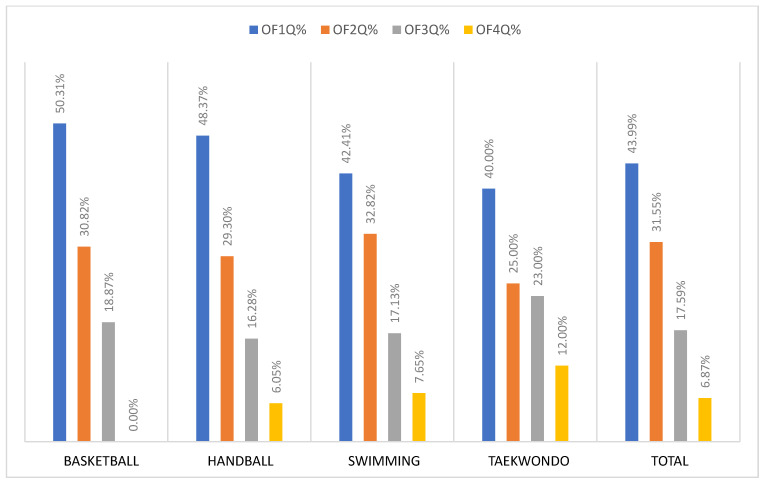
Relative observed frequencies (OF1Q%, OF2Q%, OF3Q%, OF4Q%) for each sport and total sample (RAEOS model).

Based on the results shown in Table 2 and Figure 1, it is evident that there is a statistically significant difference between observed and expected frequencies in individual sports and in total for all athletes with an error of less than 1% (*p* < 0.01), i.e., that the age of the athlete significantly determined their participation in the Youth Olympic Games in Buenos Aires 2018. Relative observed frequencies (Figure 1) show that the largest percentage of participants at the YOG 2018 were born in the first quartile, almost 44% in the total sample, while only around 7% of total participants were born in the last quartile. What interests us the most is that over 50% of young basketball players are born in the first quartile, while none of the young basketball players were born in the last quartile.

Based on the statistical analysis depicted in Table 3, a notable and statistically significant deviation exists between the anticipated and actual frequencies among first-place athletes, with a marginal error rate below 1% (*p* < 0.01). This underscores the substantial influence exerted by athletes’ ages in determining their likelihood of securing gold medals during the Youth Olympic Games held in Buenos Aires in 2018.

Based on the findings presented in Table 4, there is clear evidence indicating a relationship between athletes’ ages and their choice of sport discipline during the Youth Olympic Games in Buenos Aires in 2018. Notably, a discernible pattern emerges where a greater proportion of athletes born within the first two quartiles tend to participate in team sports such as basketball and handball, as opposed to individual sports like swimming and taekwondo. This observation highlights the influence of relative age on athletes’ sport selection, particularly in terms of the team versus individual sport distinction.

## 4. Discussion

The findings of this study contribute to the growing body of literature on the relative age effect (RAE) in youth sports, offering insights into its prevalence and implications across various sporting contexts. By introducing a novel methodological approach termed Relative Age Effect Overall Scale (RAEOS), this study extends previous research efforts aimed at understanding and addressing RAE-related challenges.

Consistent with existing literature, the results of this study confirm the presence of RAE across different sports, including basketball, handball, swimming, and taekwondo. These findings align with previous research findings that have highlighted the pervasive nature of RAE in youth sports competitions [21,22]. The proposed RAEOS methodology provides a standardized framework for analyzing RAE within each sport, facilitating comprehensive comparisons and enabling insights into the differential susceptibility to RAE across sporting disciplines. Moreover, the observed differences between team and individual sports underscore the need for tailored approaches in talent identification and development programs. While the reasons behind these differences require further investigation, previous studies have suggested various factors contributing to the differential impact of RAE across sports, including competition structures, coaching practices, and selection biases [18,23]. By elucidating these disparities, the current study highlights opportunities for optimizing talent pathways and promoting equitable opportunities for young athletes across diverse sporting contexts.

The disproportionate representation of young basketball players born in the first quartile, coupled with the absence of individuals born in the last quartile among those competing at the Youth Olympic Games (YOG) in 2018, serves as a compelling indicator of a flawed selection process biased towards those born earlier, similar to other studies [23]. The later authors have also found significant impact of the RAE on the success of youth basketball teams, indicating RAE as a key factor to consider in the early career planning of a young athlete. This pattern, which favors athletes born in the first quartile, suggests that the selection criteria employed in identifying basketball talents for elite competitions such as the YOG may inadvertently favor athletes born earlier in the selection year, thereby overlooking late-maturing individuals who may possess equal or greater potential. Such a skewed distribution can be attributed to the prevalence of the relative age effect (RAE), wherein athletes born closer to the cutoff date for age-group eligibility are perceived as more advanced or talented due to their physical maturity relative to their peers [24]. Moreover, it is plausible that this advantage was further exacerbated by the emphasis on physical attributes such as height during the selection process. Taller athletes, often associated with early maturation, may have been disproportionately favored, leading to the overrepresentation of individuals born in the first quartile. Consequently, young basketball players born later in the selection year, particularly those in the last quartile, may be unfairly disadvantaged in the talent identification process, leading to their underrepresentation in elite competitions. This phenomenon underscores the importance of implementing more equitable and inclusive selection criteria that account for individual differences in maturation rates and consider diverse aspects of athletic potential beyond physical attributes alone, thereby ensuring a fairer and more meritocratic pathway for identifying and nurturing basketball talents at the youth level.

Unlike basketball, taekwondo is an individual sport where athletes rely solely on themselves. Scientists initially hypothesized that weight categories might mitigate the impact of the Relative Age Effect (RAE) in this sport [25]. However, their conclusion did not fully support this assumption. In taekwondo, RAE was found to exist not only across age groups but also within weight categories, predominantly among seniors, albeit to a slightly lesser extent among juniors and cadets. Combining the findings from our study with existing studies, it becomes evident that RAE persists in taekwondo at the highest levels (e.g., YOG) from junior levels onward, continuing into the senior category. Whether this phenomenon is linked to accelerated limb length growth, similar to basketball players, warrants further investigation. Moreover, additional research is needed to verify differences in RAE between male and female populations using new methodologies, as previous studies have indicated a higher prevalence of RAE among males [25].

In swimming and handball, similar to taekwondo and basketball, the presence of the RAE has been documented, often influenced by the physical advantage of early-maturing athletes. This phenomenon is probably partly attributable to accelerated growth in limb length among early-maturing individuals, which can confer advantages such as enhanced strength and reach. In swimming, for instance, where events are highly dependent on physical attributes like height and arm span, and also strength demands [26], early-maturing athletes may have a significant edge in performance, influencing their selection and competitive success. Similarly, in handball, where physical prowess plays a crucial role in both attacking and defending strategies, early-maturing players may excel due to their developed physical attributes. It has also been proven that older players play more minutes and achieve better performances [27].

The implications of these findings extend to sports governance and policymaking, emphasizing the importance of addressing RAE-related challenges in competition structures and talent development programs. Efforts to enhance the fairness and inclusivity of youth sports competitions, such as the Youth Olympic Games (YOG), should consider the influence of RAE and implement strategies to mitigate its impact on athlete participation and development [3,28].

However, it is essential to acknowledge several limitations of this study. The sample, consisting of athletes participating in the YOG in Buenos Aires 2018, may not fully represent the broader population of youth athletes across different sports and regions. Additionally, while the RAEOS methodology offers a promising framework for analyzing RAE, further validation and refinement are warranted to ensure its applicability and reliability across diverse sporting contexts. Overall, this study contributes to our understanding of RAE in youth sports by introducing a novel methodological approach and providing empirical evidence of RAE across multiple sports at the YOG. By shedding light on the complex interplay between age, sport, and competition format, this research informs efforts aimed at addressing RAE-related challenges and promoting equitable opportunities for young athletes in sports participation and development programs.

## 5. Conclusions

The proposed methodological approach is a simple and applicable tool that provides opportunities for comparison and analysis of different sports and competition formats, as well as improvement of the sports talent system in the context of RAE issues. According to the conducted analyses, authors suggest that sports decision-makers improve the YOG qualification/competition system to enable fairer competition and reduce the influence of RAE on the performance and development of young athletes. The selectors of the national young sports teams or the existing qualification systems do not take into account the RAE effect, so young athletes born at the end of the age category, regardless of their potential, rarely get the opportunity to participate in major sports competitions, especially in sports with a dominant physical component. It would be interesting for future research to analyze the relationships between medal winners and other participants in the same sample in a contest of RAE and also to compare and investigate such categorization by gender. It would also be interesting to investigate the presence of RAE in other sports, both at the YOG and the Olympic Games, using the new RAEOS methodology.

## Figures and Tables

**Table 1:** A new methodological approach (RAEOS)—range of birth month per quartile for each sport.

4-Quartile Approach *						
SPO	1Q	2Q	3Q	4Q	TARM	RIPSQ
Basketball	1 m 2000–9 m 2000	10 m 2000–6 m 2001	7 m 2001–3 m 2002	4 m 2002–12 m 2003	36	9
Handball	1 m 2000–9 m 2000	10 m 2000–6 m 2001	7 m 2001–3 m 2002	4 m 2002–12 m 2003	36	9
Swimming	1 m 2000–12 m 2000	1 m 2001–12 m 2001	1 m 2002–12 m 2002	1 m 2003–12 m 2003	48	12
Taekwondo	1 m 2001–6 m 2001	7 m 2001–12 m 2001	1 m 2002–6 m 2002	7 m 2002–12 m 2002	24	6

Legend: SPO = sport, 1Q = 1Q range (months of birth), 2Q = 2Q range (months of birth), 3Q = 3Q range (months of birth), 4Q = 4Q range (months of birth), TARM = total age range in months, RIPSQ = range of individual period for 4 quartiles (number of months in each quartile). * All athletes are categorized into 4 categories in a range that is unique to each sport.

**Table 2:** Observed and expected frequencies in the number of subjects for each sport and quartile (RAEOS model). Results of Chi-square test—example of Youth Olympic Games 2018.

SPO	EFPQ	OF1Q	OF2Q	OF3Q	OF4Q	SOF	Chi-Square	*p*
Basketball	39.75	80	49	30	0	159	24.04	*p* < 0.01
Handball	53.75	104	63	35	13	215	86.01	*p* < 0.01
Swimming	245.25	416	322	168	75	981	285.42	*p* < 0.01
Taekwondo	25	40	25	23	12	100	15.92	*p* < 0.01
Total	1455	640	459	256	100	1455	457.90	*p* < 0.01

Legend: SPO = sport, EFPQ = expected frequencies per quartile, OF1Q = 1st quartile observed frequencies, OF2Q = 2nd quartile observed frequencies, OF3Q = 3rd quartile observed frequencies, OF4Q = 4th quartile observed frequencies, SOF = sum of all observed frequencies.

**Table 3:** Observed and expected frequencies in number and percentage of subjects for each quartile for the gold medalists (RAEOS model). Results of Chi-square test—example of Youth Olympic Games 2018.

Gold Medalists	EFPQ	OF1Q	OF2Q	OF3Q	OF4Q	Total	Chi-Square	*p*
All sports	15.5	33	14	10	5	62	28.97	*p* < 0.01
%	25%	53.23%	22.58%	16.13%	8.06%	100%		

Legend: EFPQ = expected frequencies per quartile, OF1Q = 1st quartile observed frequencies, OF2Q = 2nd quartile observed frequencies, OF3Q = 3rd quartile observed frequencies, OF4Q = 4th quartile observed frequencies.

**Table 4:** Relative observed frequencies, Chi-square value (χ^2^), degrees of freedom (df), and the error with which we claim that the differences between individual (I) and team (T) sports athletes in the QUARTILE variable are statistically significant (*p*).

	OF1Q%	OF2Q%	OF3Q%	OF4Q%	Total	Chi-Square	*p*
I	42.2%	32.1%	17.7%	8.0%	100%	11.98	0.007
T	49.2%	29.9%	17.4%	3.5%	100%		

## Data Availability

The data presented in this study are available on request from the corresponding author.

## References

[1] Malm C., Jakobsson J., Isaksson A. (2019). Physical activity and sports—Real health benefits: A review with insight into the public health of Sweden. Sports.

[2] Huard Pelletier V., Lemoyne J. (2022). Early Sport Specialization and Relative Age Effect: Prevalence and Influence on Perceived Competence in Ice Hockey Players. Sports.

[3] Hancock D.J., Adler A.L., Côté J. (2013). A proposed theoretical model to explain relative age effects in sport. Eur. J. Sport Sci..

[4] Babic M., Cular D., Jelaska I. (2021). Relative age effect among young Croatian taekwondo competitors. Acta Kin..

[5] Wattie N., Schorer J., Baker J. (2015). The relative age effect in sport: A developmental systems model. Sports Med..

[6] Bjerke Ø., Lorås H., Pedersen A.V. (2023). Constituent Year Effects and Performance in Alpine Skiing Junior World Championships. Sports.

[7] Cular D., Miletic A., Babic M. (2024). The prevalence of Constituent Year effect in Youth Olympic Games: Implications for talent identification and development in basketball. Acta Kin..

[8] Helsen W.F., Van Winckel J., Williams A.M. (2005). The relative age effect in youth soccer across Europe. J. Sports Sci..

[9] Steidl-Müller L., Hildebrandt C., Raschner C., Müller E. (2019). Challenges of talent development in alpine ski racing: A narrative review. J. Sports Sci..

[10] Babic M., Macan I., Beslija T., Kezic A., Tomljanovic M., Subasic L., Cular D. (2022). Relative age effect and gender differentiation within sport-a systematic review. Acta Kin..

[11] Pino-Ortega J., Gómez-Carmona C.D., Nakamura F.Y., Rojas-Valverde D. (2022). Setting kinematic parameters that explain youth basketball behavior: Influence of relative age effect according to playing position. J. Strength Cond. Res..

[12] Faber I.R., Liu M., Cece V., Jie R., Martinent G., Schorer J., Elferink-Gemser M.T. (2020). The interaction between within-year and between-year effects across ages in elite table tennis in international and national contexts—A further exploration of relative age effects in sports. High Abil. Stud..

[13] Pedersen A.V., Aune T.K., Dalen T., Lorås H. (2022). Variations in the relative age effect with age and sex, and over time—Elite-level data from international soccer world cups. PLoS ONE.

[14] Smith K.L., Weir P.L., Till K., Romann M., Cobley S. (2018). Relative age effects across and within female sport contexts: A systematic review and meta-analysis. Sports Med..

[15] Tribolet R., Watsford M.L., Coutts A.J., Smith C., Fransen J. (2019). From entry to elite: The relative age effect in the Australian football talent pathway. J. Sci. Med. Sport.

[16] de la Rubia A., Lorenzo-Calvo J., Lorenzo A. (2020). Does the relative age effect influence short-term performance and sports career in team sports? A qualitative systematic review. Front. Psychol..

[17] de la Rubia Riaza A., Lorenzo Calvo J., Mon-López D., Lorenzo A. (2020). Impact of the relative age effect on competition performance in basketball: A qualitative systematic review. Int. J. Environ. Res. Public Health.

[18] Cobley S., Baker J., Wattie N., McKenna J. (2009). Annual age-grouping and athlete development. Sports Med..

[19] Cumming S., Pi-Rusiñol R., Rodas G., Drobnic F., Rogol A.D. (2024). The validity of automatic methods for estimating skeletal age in young athletes: A comparison of the BAUSport ultrasound system and BoneXpert with the radiographic method of Fels. Biol. Sport.

[20] Sweeney L., Cumming S.P., MacNamara Á., Horan D. (2023). A tale of two selection biases: The independent effects of relative age and biological maturity on player selection in the Football Association of Ireland’s national talent pathway. Int. J. Sports Sci. Coach..

[21] Delorme N., Boiché J., Raspaud M. (2010). Relative age effect in elite sports: Methodological bias or real discrimination?. Eur. J. Sport Sci..

[22] Schorer J., Cobley S.P., Busch D., Brautigam H., Baker J. (2009). Influences of competition level, gender, player nationality, career stage, and playing position on relative age effects. Scand. J. Med. Sci. Sports.

[23] Rubajczyk K., Świerzko K., Rokita A. (2017). Doubly disadvantaged? The relative age effect in Poland’s basketball players. J. Sports Sci. Med..

[24] Musch J., Grondin S. (2001). Unequal competition as an impediment to personal development: A review of the relative age effect in sport. Dev. Rev..

[25] Albuquerque M.R., Fukuda D.H., Da Costa V.T., Lopes M.C., Franchini E. (2016). Do weight categories prevent athletes from the relative age effect? a meta-analysis of combat sports. Sport Sci. Health.

[26] Lorenzo-Calvo J., de la Rubia A., Mon-López D., Hontoria-Galán M., Marquina M., Veiga S. (2021). Prevalence and impact of the relative age effect on competition performance in swimming: A systematic review. Int. J. Environ. Res. Public Health.

[27] Rubia A.D.L., Bjørndal C.T., Sánchez-Molina J., Yagüe J.M., Calvo J.L., Maroto-Izquierdo S. (2020). The relationship between the relative age effect and performance among athletes in World Handball Championships. PLoS ONE.

[28] Baker J., Schorer J., Cobley S. (2010). Relative age effects: An inevitable consequence of elite sport?. Sports Med..

